# Best‐Performing Dietary Supplementation Levels of Two Exogenous Proteases for Improving Growth Performance and Nutrient Digestibility in Juvenile *Penaeus vannamei* Raised Under Clear and Green‐Water Conditions

**DOI:** 10.1155/anu/3542300

**Published:** 2026-11-04

**Authors:** Alberto J. P. Nunes, Thiago Soligo, Ester Santigosa, Caio G. D. Gomes

**Affiliations:** ^1^ LABOMAR – Instituto de Ciências do Mar Universidade Federal do Ceará Avenida da Abolição, 3207 – Meireles Fortaleza Ceará 60.165-081 Brazil ufc.br; ^2^ dsm-firmenich Brasil Rodovia Raposo Tavares, Km 26,150 – Moinho Velho Cotia São Paulo 06.707-000 Brazil; ^3^ dsm-firmenich Wurmisweg 576 4303 Kaiseraugst Switzerland

**Keywords:** amino acid bioavailability, exogenous enzymes, plant-protein diets, protein utilization

## Abstract

This study evaluated the best‐performing dietary inclusion levels of two commercial exogenous proteases, Ronozyme ProAct and ProAct 360 (dsm‐firmenich Animal Nutrition and Health, Heerlen, The Netherlands), for improving growth performance and nutrient digestibility in juvenile *Penaeus vannamei* reared under indoor and outdoor conditions. The study comprised two dose‐response growth trials conducted under contrasting indoor clear‐water and outdoor green‐water production systems, followed by two digestibility assays using diets containing either standard (33.66%) or high (51.82%) soybean meal (SBM) inclusion levels. In the indoor trial (175 shrimp/m^2^), the highest final body weights were achieved with 800 mg/kg Ronozyme ProAct (8.00 ± 0.59 g), compared with 7.48 ± 0.71 g in the control. In the outdoor trial (70 shrimp/m^2^), supplementation with 400 mg/kg Ronozyme ProAct produced the highest final body weight (10.46 ± 0.64 g), weekly growth rate (1.11 ± 0.17 g), and yield (767 ± 124 g/m^2^), significantly outperforming the control (8.60 ± 1.18 g; 0.81 ± 0.13 g/week; 558 ± 89 g/m^2^). The digestibility assays revealed no significant differences in growth performance but showed consistent improvements in apparent digestibility coefficients (ADCs). In standard SBM diets, crude protein (CP) digestibility increased from 67.80 ± 6.96% in the control to 78.16 ± 2.31% with Ronozyme ProAct and 75.27 ± 8.38% with ProAct 360, while gross energy (GE) ADC reached 74.56% with ProAct 360, compared with 58.42% in the control. In high SBM diets, supplementation with Ronozyme ProAct increased CP digestibility from 72.40 ± 6.56 to 81.34 ± 1.54%, ether extract (EE) ADC rose from 77.41 ± 5.73 to 84.87 ± 0.84%, and total amino acid digestibility increased from 78.87 ± 5.24 to 86.76 ± 0.99%, with lysine and methionine digestibility exceeding 88%. Overall, exogenous proteases enhanced nutrient utilization, particularly in diets rich in plant ingredients. The best‐performing supplementation levels differed between the two culture systems evaluated, with higher supplementation levels required indoors (800 mg/kg Ronozyme ProAct and 200 mg/kg ProAct 360) and lower levels outdoors (400 mg/kg and 100 mg/kg, respectively). These findings demonstrate the value of exogenous proteases for improving nutrient digestibility and supporting shrimp performance in practical diets containing moderate to high levels of SBM.

## 1. Introduction

In recent years, a growing trend has emerged in Latin America toward the formulation of grower diets for marine shrimp that incorporate low levels of fishmeal (<5% of the diet, as‐is) in combination with 50% or more soybean meal (SBM) [1]. This shift is primarily driven by economic pressures and the need to reduce dependence on marine resources. Although several studies have indicated that juvenile *Penaeus vannamei* can achieve adequate growth performance when reared on diets containing high proportions of plant‐derived ingredients [2–7], high‐SBM diets are prone to nutritional limitations, including variable digestibility, reduced availability of indispensable amino acids, and the presence of antinutritional factors. Extrusion technology has facilitated the use of SBM by reducing some of these limitations [8, 9].

The broader adoption of digestible‐based and least‐cost formulation strategies has allowed nutritionists to identify specific limitations of high‐SBM diets in *P. vannamei*, including inadequate digestible methionine and lysine supply, suboptimal mineral bioavailability, and reduced protein digestibility due to antinutritional factors [10]. Concurrently, the increasing use of automated mechanical feeders across the region has potentially enhanced the effectiveness of dietary supplementation with crystalline amino acids (CAAs), trace minerals, attractants, and various functional feed additives, as frequent feed delivery can reduce nutrient leaching into the water column [11–13].

Despite these advances, there remains a need to improve feed conversion efficiency while reducing nitrogen excretion and its environmental load in shrimp production systems. Reported protein apparent digestibility coefficients (ADCs) for SBM in *P. vannamei* vary significantly (96.9 ± 1.3%, Cruz‐Suárez et al. [14]; 93.6 ± 3.9–99.8 ± 1.3%, Zhou et al. [15]; and 45%–98%, Galkanda‐Arachchige et al. [16]). Early work by Divakaran et al. [17] demonstrated that this wide variability is, in part, methodological, showing that both the SBM inclusion level and chromic oxide marker concentration significantly influence calculated ADCs, occasionally yielding unrealistically high values (>100%). More recently, Lemos et al. [18] also demonstrated that published ADC values for SBM are likely inflated. Using a more physiologically robust graded–inclusion approach, the authors reported lower but more realistic indispensable amino acid digestibility values (76%–88%), suggesting that the digestible amino acid contribution of SBM has been systematically overestimated.

In practical feed formulations, progressive increases in the dietary inclusion of SBM often result in reductions in dry matter (DM) and protein digestibility, largely due to decreased trypsin, amylase, and lipase activities associated with SBM’s antinutritional factors [19]. Consistently, Nunes and Masagounder [20] reported that protein ADC declined from 88.6 ± 2.1 to 81.1 ± 1.7% when SBM increased from 20% to 45% (% of the diet, as‐is), respectively. These values demonstrate that digestibility challenges are particularly relevant for *P. vannamei*.

In parallel, dietary supplementation with exogenous proteases has gained attention as a strategy to enhance the nutritional quality of plant‐based and low–fishmeal diets for *P. vannamei*. For this species, studies have documented improvements in crude protein (CP) and amino acid digestibility, feed efficiency, gut morphology, and immune status following dietary supplementation with proteases, particularly under conditions where antinutritional factors restrict nutrient availability [21–23]. However, the literature remains inconsistent. Coelho et al. [24] reported no improvements in growth performance or nutrient digestibility when protease was supplemented to a fishmeal‐free diet. These contradictory findings show the need to clarify how ingredient composition, enzyme–substrate interactions, and baseline dietary adequacy influence protease efficacy in shrimp feeds.

Given these contrasting results, there remains a critical need to define the conditions and doses at which exogenous proteases improve nutrient digestibility and growth performance in *P. vannamei*. The present study evaluated the dose‐response relationships of two proteases differing in strain origin and catalytic activity under clear‐ and green‐water conditions and with moderate and high SBM inclusion levels.

## 2. Materials and Methods

### 2.1. Experimental Design

The study comprised four independent trials designed to determine the effects of two commercial proteases, Ronozyme ProAct (RZ) and ProAct 360 (PA) (dsm‐firmenich Animal Nutrition and Health, Heerlen, The Netherlands), on the growth performance and nutrient digestibility of juvenile whiteleg shrimp under different rearing conditions and dietary formulations (Table 1). The principal objective was to identify the best‐performing dietary supplementation level of each enzyme for maximizing shrimp growth performance and nutrient digestibility.

**Table 1:** Summary of the experimental design and culture conditions for the growth performance and digestibility trials.

Characteristics	Dose response		In vivo digestibility	
	Indoor	Outdoor	Standard SBM diet	High SBM diet
Objective	Determine the best‐performing protease dose without natural food	Determine the best‐performing protease dose with natural food	Determine protease effect on digestibility (standard SBM)	Determine protease effect on digestibility (high SBM)
Tank system	Clear‐water, recirculation	Green‐water, static	Clear‐water, recirculation	Clear‐water, recirculation
Stocking density	175 shrimp/m^2^	70 shrimp/m^2^	60 shrimp/m^2^	60 shrimp/m^2^
Initial shrimp body weight	1.04 ± 0.08 g	0.51 ± 0.03 g	7.79 ± 0.55 g	6.99 ± 0.55 g
Number of treatments	7	7	3	3
Number of replicates	14	7	14	14
Trial duration (days)	78	70	64	70

The two commercial proteases were supplemented at different inclusion levels to account for variations in their specific activity and formulation characteristics. Ronozyme ProAct (RZ) is a serine protease preparation manufactured using a genetically modified strain of *Bacillus licheniformis* (DSM 19670). The enzyme was provided as a thermostable‐coated granulate with a declared minimum activity of 75,000 PROT/g. ProAct 360 (PA) is produced by a genetically modified strain of *B. licheniformis* (DSM 33099) and was supplied as a coated granulated formulation with a minimum guaranteed activity of 600,000 NFP/g. The activities of the two products are expressed in different units because they are determined using different analytical methods developed specifically for each enzyme product. Enzyme activities in the experimental diets were verified by the manufacturer’s accredited laboratory using method SOY‐101/04E for Ronozyme ProAct (reported as PROT/kg) and method PROT2G_04_004_01 for ProAct 360 (reported as NFP/kg). In this study, dietary supplementation levels ranged from 200 to 800 mg/kg for RZ and 100 and 200 mg/kg for PA, thereby providing a graded range of dietary protease activities.

### 2.2. Dose‐Response Experiments (Phases 1 and 2)

Postlarvae 10 (PL10) of *P. vannamei* were purchased from a commercial hatchery (Aquatec Aquacultura Ltda., Canguaretama, Brazil) and transported in plastic bags to the laboratory. Shrimp were raised in the lab from PL until juvenile size prior to experiments. Separate batches of shrimp were used for each experimental phase according to the target initial body weights. The first trial was conducted in a clear‐water tank system (98 tanks with 14 replicates) in the absence of natural food. Tanks were operated under continuous water recirculation with a high shrimp stocking density (175 shrimp/m^2^). The outdoor trial (56 tanks and seven replicates) was conducted in a green‐water static system under moderate stocking density (70 shrimp/m^2^) with a potential contribution from natural productivity to shrimp nutrition. Natural food was developed by fertilizing culture water prior to shrimp stocking using a fermented mixture of a commercial probiotic and sugar‐cane molasses [25]. The shrimp initial body weights in these trials were 1.04 ± 0.08 g (indoor) and 0.51 ± 0.03 g (outdoor) and were reared for 78 and 70 days, respectively. The two growth trials were designed to represent contrasting culture systems rather than to isolate the effects of individual environmental variables. Consequently, differences between trials (e.g., water management, natural productivity, stocking density, initial shrimp body weight, and culture duration) were inherent to the experimental design and should be interpreted collectively.

Seven practical diets were formulated using a common ingredient and nutrient matrix and differed only in protease type and inclusion level. A control diet (CTRL) without protease served as the baseline. Four diets were supplemented with Ronozyme ProAct (RZ) at 200, 400, 600, and 800 mg/kg, corresponding to 0.020% (RZ20; 8,653 ± 76 PROT/kg, *n* = 2), 0.040% (RZ40; 19,755 ± 1068 PROT/kg), 0.060% (RZ60; 34,745 ± 2666 PROT/kg), and 0.080% (RZ80; 47,520 ± 1414 PROT/kg), while two diets contained ProAct 360 (PA) at 100 and 200 mg/kg, equivalent to 0.010% (PA10; 41,195 ± 35 NFP/kg) and 0.020% (PA20; 76,720 ± 552 NFP/kg). All dose‐response diets contained 30.57% SBM (as‐is basis), representing the basal practical formulation used in the growth trials. Experimental diets were isonitrogenous and isolipidic and were formulated to represent a practical shrimp feed (Table 2).

**Table 2:** Ingredient composition (% as‐is basis), analyzed proximate composition, and measured protease activity of diets used in the dose‐response growth trials.

Ingredients	Diets/ingredient composition (%, as‐is basis)						
	CTRL	RZ20	RZ40	RZ60	RZ80	PA10	PA20
Wheat flour^a^	36.00	36.00	36.00	36.00	36.00	36.00	36.00
Soybean meal^b^	30.57	30.57	30.57	30.57	30.57	30.57	30.57
Poultry by‐product meal^c^	7.00	7.00	7.00	7.00	7.00	7.00	7.00
Salmon by‐product meal^d^	7.00	7.00	7.00	7.00	7.00	7.00	7.00
Cassava starch^e^	4.50	4.48	4.46	4.44	4.42	4.49	4.48
Salmon oil^f^	3.09	3.09	3.09	3.09	3.09	3.09	3.09
MSP^g^	2.89	2.89	2.89	2.89	2.89	2.89	2.89
Squid meal^h^	2.00	2.00	2.00	2.00	2.00	2.00	2.00
Calcium carbonate^i^	1.19	1.19	1.19	1.19	1.19	1.19	1.19
Soy lecithin, oil	1.16	1.16	1.16	1.16	1.16	1.16	1.16
Magnesium sulfate	0.98	0.98	0.98	0.98	0.98	0.98	0.98
Potassium chloride	0.78	0.78	0.78	0.78	0.78	0.78	0.78
Salt, coarse	0.77	0.77	0.77	0.77	0.77	0.77	0.77
L‐lysine, 54.6%^j^	0.67	0.67	0.67	0.67	0.67	0.67	0.67
Synthetic binder^k^	0.50	0.50	0.50	0.50	0.50	0.50	0.50
Vitamin‐mineral premix^l^	0.30	0.30	0.30	0.30	0.30	0.30	0.30
L‐threonine, 98.5%^m^	0.27	0.27	0.27	0.27	0.27	0.27	0.27
DL‐methionine, 99%^n^	0.19	0.19	0.19	0.19	0.19	0.19	0.19
Choline chloride, 60%^o^	0.14	0.14	0.14	0.14	0.14	0.14	0.14
Ronozyme ProAct^p^	—	0.020	0.040	0.060	0.080	—	—
ProAct 360^q^	—	—	—	—	—	0.010	0.020
Proximate composition (%, as‐is)							
Moisture	12.10	11.97	12.01	11.60	10.26	11.60	9.91
Crude protein	30.85	30.82	30.96	30.83	30.98	30.73	30.81
Ether extract	7.37	7.59	7.59	7.39	7.25	7.59	7.32
Total fiber	1.28	1.44	1.33	1.61	1.40	1.62	1.92
Ash	7.56	8.18	7.99	8.45	8.51	8.92	9.64
Calcium	0.84	0.77	0.64	0.87	0.95	0.89	0.91
Phosphorus	1.19	0.97	0.86	1.22	1.20	1.21	1.22
Measured protease activity^r^ (PROT or NFP × 10^3^/kg diet)	—	8.6 ± 0.1	19.8 ± 1.1	34.7 ± 2.7	47.5 ± 1.4	41.2 ± 0.1	76.7 ± 0.6

Abbreviations: CTRL, control; PA, ProAct 360; RZ, Ronozyme ProAct.

^a^11.92% crude protein (CP), 1.20% ether extract (EE), 0.30% total fiber, 0.60% ash, 13.23% moisture, 0.18% methionine (Met), 0.44% Met + Cysteine (M + C), 0.26% lysine (Lys), and 0.30% threonine (Thr).

^b^Bunge Alimentos S.A. (Luiz Eduardo Magalhães, Brazil). 45.26% CP, 2.28% EE, 6.80% fiber, 6.10% ash, 10.94% moisture, 0.58% Met, 1.22% M + C, 2.75% Lys, and 1.74% Thr.

^c^BRF S.A. (Capinzal, Brazil). Poultry by‐product meal, low‐ash. 69.79% CP, 13.66% EE, 1.40% fiber, 8.48% ash, 4.08% moisture, 1.49% Met, 2.35% M + C, 4.36% Lys, and 2.85% Thr.

^d^Fiordo Austral Group (Puerto Montt, Chile). 63.86% CP, 9.30% EE, 0.14% fiber, 16.20% ash, 10.58% moisture, 1.65% Met, 2.35% M + C, 4.19% Lys, and 2.35% Thr.

^e^2.47% CP.

^f^Fiordo Austral Group (Puerto Montt, Chile). 99.52% total lipids, 18.34% LOA, 5.70% LNA, 0.26% ARA, 3.25% EPA, 4.12% DHA, 13.60% total n‐3 PUFA, and 19.98% total n‐6 PUFA.

^g^Monobasic sodium phosphate. 0.60% calcium, 20.70% phosphorous, and 14.12% available phosphorous.

^h^83.13% CP, 5.65% EE, 9.75% moisture, 2.34% Met, 3.17% M + C, 5.06% Lys, and 3.33% Thr.

^i^HiWhite 280, precipitated calcium carbonate. Tansan Industria Quimica Ltda. (Pedra do Indaiá, Brazil).

^j^Biolys, Evonik Operations GmbH (Hanau, Germany). L‐lysine 54.6%.

^k^Nutri‐Bind Aqua Dry, Adisseo France SAS (Antony, France). Synthetic pellet binder consisting of modified urea (polymethylolcarbamide).

^l^Rovimix 2050 VM 10 L. DSM Produtos Nutricionais Brasil Ltda. (São Paulo, Brazil). Guarantee levels per kg of product: vitamin A, 3,500,000 IU; vit. D3, 1,500,000 IU; vit. E, 75,000 IU; vit. K3, 15 g; vit. B1, 12.5 g; vit. B2, 10 g; vit. B6; 12.5 mg; vit. B16, 10 µg; folic acid, 5000 mg; nicotinic acid, 50 g; pantothenic acid, 40 mg; biotin, 500 mg; vit. C, 100 g; Cu, 12.5 g; Fe, 15 g; I, 500 mg; Mn, 15 g; Se, 175 mg; and Zn, 50 g. Sample supplied by the contracting party.

^m^ThreAMINO, Evonik Operations GmbH (Hanau, Germany). L‐threonine 98.5%.

^n^MetAMINO, Evonik Operations GmbH (Hanau, Germany). DL‐methionine, feed grade 99%.

^o^60% of active choline. Zouping Jujia Choline Industrial Co., Ltd. (Handian Industrial Zone, Zouping County, Shandong, China).

^p^DSM Produtos Nutricionais Brasil S.A. (São Paulo, Brazil). Minimum guaranteed enzyme activity of 75,000 PROT/g. PROT, protease unit.

^q^DSM Produtos Nutricionais Brasil S.A. (São Paulo, Brazil). Minimum guaranteed enzyme activity is 600,000 NFP/g. NFP, novo protease units.

^r^Values presented as mean ± standard deviation (*n* = 2).

### 2.3. Digestibility Experiments (Phases 3 and 4)

The final two phases evaluated the effects of protease supplementation on diet digestibility using a clear‐water recirculating system. Both assays used 44 rectangular 61‐L tanks with 14 replicates per treatment and a stocking density of 60 shrimp/m^2^. Detailed specifications of the digestibility tanks and recirculating system are provided by Vieira et al. [26] and Nunes et al. [27]. Initial shrimp body weights averaged 7.79 ± 0.55 g and 6.99 ± 0.55 g, and the rearing periods lasted 64 and 70 days, respectively. The primary objective of these trials was to quantify the ADCs of nutrients and amino acids. Growth performance variables were recorded for descriptive purposes only and were not intended as response criteria due to the feeding strategy used to ensure sufficient feces collection.

Two sets of diets were formulated containing different SBM levels (Table 3). The first set represented a standard SBM level (33.66% SBM), typical of commercial grower shrimp feeds, while the second incorporated a high SBM level (51.82% SBM), designed to assess enzyme efficacy in diets with elevated plant protein content. Each set consisted of three treatments: a CTRL diet without enzyme addition and two diets supplemented with each protease type, RZ at 400 mg/kg and PA at 100 mg/kg. The standard SBM diets (CTRL, RZ40, and PA10) were formulated with CP levels of 33.15%, 32.80%, and 32.72% (% of the diet, DM basis), respectively (Table 4). The high SBM diets (CTRL, RZ40, and PA10) were formulated with higher protein content, reaching 40.22%, 40.19%, and 40.16% CP, respectively.

**Table 3:** Ingredient composition (% as‐is basis) of experimental diets used in the digestibility trials.

Ingredients	Standard SBM diets			High SBM diets		
	CTRL	RZ40	PA10	CTRL	RZ40	PA10
Soybean meal^a^	33.66	33.66	33.66	51.82	51.82	51.82
Wheat flour^b^	35.00	35.00	35.00	23.79	23.75	23.78
Salmon meal^c^	7.00	7.00	7.00	4.00	4.00	4.00
Corn gluten meal^d^	—	—	—	5.42	5.42	5.42
Cassava starch^e^	6.24	6.20	6.23	—	—	—
Poultry by‐product meal^e^	4.00	4.00	4.00	2.00	2.00	2.00
Salmon oil^e^	3.43	3.43	3.43	2.54	2.54	2.54
Sodium monophosphate^e^	2.44	2.44	2.44	1.81	1.81	1.81
Calcium carbonate^e^	1.57	1.57	1.57	1.69	1.69	1.69
Magnesium sulfate	1.33	1.33	1.33	1.77	1.77	1.77
Soy lecithin oil	0.98	0.98	0.98	1.21	1.21	1.21
Potassium chloride	0.90	0.90	0.90	1.15	1.15	1.15
Salt coarse	0.83	0.83	0.83	0.69	0.69	0.69
Squid meal^f^	—	—	—	0.50	0.50	0.50
L‐lysine^e^	0.67	0.67	0.67	—	—	—
Chromium III oxide^g^	0.50	0.50	0.50	0.50	0.50	0.50
Synthetic binder^e^	0.50	0.50	0.50	0.50	0.50	0.50
Vitamin‐mineral premix^e^	0.30	0.30	0.30	0.30	0.30	0.30
DL‐methionine^e^	0.26	0.26	0.26	0.15	0.15	0.15
L‐threonine^e^	0.24	0.24	0.24	0.01	0.01	0.01
Choline chloride, 60%^e^	0.15	0.15	0.15	0.15	0.15	0.15
Ronozyme ProAct^e^	—	0.040	—	—	0.040	—
ProAct 360^e^	—	—	0.010	—	—	0.010

Abbreviation: SBM, soybean meal.

^a^Bunge Alimentos S.A. (Luiz Eduardo Magalhães, Brazil). 47.57% crude protein (CP), 1.82% ether extract (EE), 3.02% total fiber, 6.86% ash, 8.55% moisture, 0.61% methionine (Met), 1.28% methionine + cysteine (M + C), 2.92% lysine (Lys), and 1.82% threonine (Thr).

^b^10.61% CP, 1.06% EE, 0.18% total fiber, 0.67% ash, 13.18% moisture, 0.15% Met, 0.36% M + C, 0.21% Lys, and 0.24% Thr.

^c^Fiordo Austral Group (Puerto Montt, Chile). 67.08% CP, 10.88% EE, 0.13% total fiber, 14.07% ash, 7.55% moisture, 1.69% Met, 2.35% M + C, 4.47% Lys, and 2.45% Thr.

^d^Protenose, Ingredion Brasil Ingredientes Industriais Ltda. (Cabo de Santo Agostino, Brazil). 56.28% CP, 7.82% EE, 0.79% total fiber, 1.62% ash, 10.79% moisture, 1.24% Met, 2.22% M + C, 1.03% Lys, and 1.86% Thr.

^e^Described previously in Table 2.

^f^Peruvian Sea Food S.A. (Paita, Peru). 80.18% CP, 4.28% EE, 2.37% total fiber, 7.44% ash, 6.81% moisture, 2.25% Met, 3.08% M + C, 5.03% Lys, and 3.23% Thr.

^g^Sigma–Aldrich Co. (St. Louis, USA), 50‐micron powder, 98%.

**Table 4:** Analyzed proximate composition and amino acid profile (% dry matter basis) of diets used in the digestibility trial.

Nutrient or energy	Standard SBM diets			High SBM diets		
	CTRL	RZ40	PA10	CTRL	RZ40	PA10
Crude protein (CP)	33.15	32.80	32.72	40.22	40.19	40.16
Ether extract	7.47	7.51	7.92	7.04	7.44	6.90
Crude fiber	3.03	2.61	2.66	2.64	2.63	2.64
Ash	10.78	10.81	10.69	9.37	9.35	9.37
Gross energy (MJ/kg)	17.74	17.89	18.09	18.38	17.45	19.50
Essential amino acids (EAAs)						
Arginine (Arg)	1.95	2.04	2.10	2.42	2.65	2.21
Histidine (His)	0.82	0.83	0.83	0.86	0.99	0.85
Isoleucine (Ile)	1.28	1.33	1.32	1.67	1.67	1.61
Leucine (Leu)	2.31	2.31	2.30	3.13	3.11	2.96
Lysine (Lys)	2.04	2.27	2.23	1.93	1.92	1.91
Methionine (Met)	0.73	0.81	0.82	0.66	0.67	0.64
Met + Cys	1.21	1.30	1.34	1.16	1.26	1.13
Phenylalanine (Phe)	1.49	1.61	1.58	1.86	2.35	1.76
Threonine (Thr)	1.45	1.73	1.76	1.47	1.57	1.40
Valine (Val)	1.42	1.44	1.44	1.76	1.81	1.68
Nonessential amino acids (NEAAs)						
Alanine (Ala)	1.45	1.47	1.47	1.93	1.98	1.81
Aspartic acid (Asp)	2.83	2.63	2.77	3.70	3.63	3.45
Cysteine (Cys)	0.48	0.49	0.52	0.49	0.59	0.49
Glycine (Gly)	1.80	1.89	1.93	1.76	1.81	1.64
Glutamic acid (Glu)	6.00	5.97	6.15	7.09	7.01	6.55
Hydroxyproline (Hyd)	0.01	0.01	0.01	0.01	0.01	0.01
Proline (Pro)	1.77	1.73	1.75	2.25	3.76	2.08
Serine (Ser)	1.55	1.51	1.56	1.91	1.99	1.75
Taurine (Tau)	0.01	0.01	0.01	0.06	0.08	0.01
Tyrosine (Tyr)	1.08	1.03	1.06	1.44	1.61	1.26
Sum EAA^a^	13.49	14.38	14.38	15.76	16.74	15.02
Sum NEAA	16.99	16.75	17.22	19.21	20.87	17.79
EAA + NEAA	30.48	31.13	31.61	34.97	37.61	32.81

^a^Tryptophan not included.

Experimental diets were prepared using pilot‐scale feed manufacturing equipment. Dry ingredients were first ground to achieve a uniform particle size of less than 300 microns. After weighing, feed additives (vitamin‐mineral premix, amino acids, enzymes, binder, etc.) and chromium III oxide (for digestibility diets) were premixed in a Y‐mixer with a portion of the dry ingredients before combining with the macro ingredients. Once mixed completely, they were added to a planetary mixer containing all liquid and dried raw materials. For mixing, an average of 5014.0 ± 22.0 g of tap water at 29.0 ± 0.9°C was added for every 15 kg of feed mash produced. This increased the moisture content of the feed mash to 33.2 ± 0.8%. The moist feed dough was broken into small particles, which were introduced into the pelleting machine adjusted to operate at 95°C. This increased the temperature of the feed mash and the pellets exiting the pelleting machine to 41.0 ± 2.0 and 61.5 ± 1.0°C, respectively. Once pellets were cut, they were subjected to steam‐conditioning for 3 min and subsequently dried to a final moisture content of 8.0%–10.0%. Feed pellets measured 1.69 ± 0.09 mm (mean ± standard deviation; *n* = 90) in diameter by 4.34 ± 0.36 mm (*n* = 90) in length.

### 2.4. Feeding, Collection of Fecal Material, and Chemical Analysis

In the indoor and outdoor tank systems, shrimp were fed manually four times daily (07:00, 10:00, 13:00, and 16:00 h) throughout the study using feeding trays. Shrimp were fed to the maximum feed intake model proposed by Nunes and Parsons [28], with daily feed allowances reduced by 28.8% [29] to avoid overfeeding. For the digestibility trials, shrimp were fed in excess three times per day (07:00, 13:00, and 16:00 h) to maximize feces production. Fecal material was collected 1 h after feeding through siphoning, rinsed with distilled water, and stored frozen until freeze‐drying and subsequent chemical analysis [26]. For chemical analyses, fecal samples from three to four replicate tanks receiving the same dietary treatment were composited prior to analysis to generate four independent composite samples per treatment. Composite sampling was adopted to optimize laboratory analyses while maintaining adequate analytical replication for digestibility determinations. In all studies, water temperature, pH, and salinity were measured daily in each tank. Continuous bottom aeration was provided using blowers to maintain dissolved oxygen concentrations near saturation throughout the culture period. Chemical analyses of experimental diets and feces followed the procedures described by Nunes et al. [27]. Moisture, CP, ether extract (EE), ash, crude fiber, and gross energy (GE) were determined according to AOAC [30] and AOCS [31] methods. Amino acid profile was determined by ion‐exchange chromatography [32, 33], while chromium concentrations were measured by inductively coupled plasma triple quadrupole mass spectrometry (ICP‐QQQ).

### 2.5. Growth Performance, ADCs, and Statistical Analysis

At the end of the culture period, shrimp were captured and individually weighed. Growth performance variables, including survival (%), final body weight (g), weekly weight gain (g), gained yield (g/m^2^), feed conversion ratio (FCR), and apparent feed intake (AFI, g of feed delivered per stocked shrimp), were determined following the calculations presented in Nunes et al. [27]. ADCs of DM, CP, EE, GE, and AAs were determined using chromic oxide as an inert marker according to the equation provided by Cho et al. [34]. Growth performance variables and ADCs were subjected to one‐way analysis of variance (ANOVA), followed by Tukey’s multiple‐comparison test when significant differences were detected. The probability level for significance was set at *α* = 0.05. Statistical analyses were carried out using the IBM SPSS Statistics version 23.0 (IBM Corp., Chicago, IL, USA).

## 3. Results

### 3.1. Enzyme Activity and Diet Chemical Composition

For the dose‐response trial, the proximate composition of the diets was comparable among treatments, averaging 30.85 ± 0.09% CP and 7.44 ± 0.14% EE. Measured protease activity ranged from 8.6 to 47.5 PROT/g in RZ‐supplemented diets and from 41.2 to 76.7 NFP/g in PA‐supplemented diets, confirming the expected gradient of enzyme activity for the dose‐response evaluation.

In the standard SBM diets used in the digestibility assay, total EE content ranged from 7.47% to 7.92 %, while crude fiber varied between 2.61% and 3.03% (Table 4). Ash content remained stable, between 10.69% and 10.81 %, and GE values ranged from 17.74 to 18.09 MJ/kg, indicating consistent nutrient composition among the three diets. The sum of essential amino acids (EAAs) was 13.49% in CTRL and 14.38% in both RZ40 and PA10, with total amino acids (EAA + non‐EAA [NEAA]) ranging from 30.48% to 31.61%. Within this set, lysine (2.04%–2.27%), methionine (0.73%–0.82%), and methionine + cysteine (1.21%–1.34%) levels were close to the established amino acid requirements for *P. vannamei*. Glutamic acid (5.97%–6.15%), aspartic acid (2.63%–2.83%), and proline (1.73%–1.77%) were the predominant NEAAs. The similarity among CTRL, RZ40, and PA10 indicates that the inclusion of protease enzymes did not affect the proximate or amino acid composition within the standard SBM diet group.

In the high SBM diets, total EE levels varied from 6.90% to 7.44%, while crude fiber values remained uniform at 2.63%–2.64%. Ash content was slightly reduced compared with the standard SBM diets, ranging from 9.35% to 9.37%, consistent with the higher proportion of plant‐based ingredients. GE varied between 17.45 and 19.50 MJ/kg, confirming the maintenance of near‐isoenergetic formulations. The sum of EAA increased to 15.76% (CTRL), 16.74% (RZ40), and 15.02% (PA10), while total amino acids (EAA + NEAA) ranged from 32.81% to 37.61%, reflecting the higher protein concentration. Within this set, arginine (2.21%–2.65%) and leucine (2.96%–3.13%) were elevated, consistent with the amino acid profile of SBM. In contrast, lysine (1.91%–1.93%) and methionine (0.64%–0.67%) were reduced relative to the standard diets, illustrating the dilution of sulfur amino acids and lysine in high‐SBM formulations. Among NEAA, glutamic acid (6.55%–7.09%), aspartic acid (3.45%–3.70%), and proline (2.08%–3.76%) were predominant, with the substantial increase in proline reflecting the higher contribution of SBM protein.

Comparatively, the transition from the standard to the high SBM diet set resulted in a 7%–8% point increase in CP and a proportional rise in total AAs, while energy and fiber contents remained stable. The compositional uniformity within each diet set demonstrates that the protease treatments (RZ40 and PA10) were introduced without altering the baseline nutritional profile. Therefore, any differences observed in digestibility or performance responses can be attributed to the enzymatic treatments rather than to inherent variations in diet composition.

### 3.2. Dose Response

In the indoor clear‐water culture system (175 shrimp/m^2^), temperature, salinity, and pH reached 28.1 ± 0.6°C, 12.0 ± 2.3 ppt, and 8.2 ± 0.2, respectively. No significant differences (*p* > 0.05) were detected among diets for final shrimp survival, gained yield, weekly growth, AFI, or FCR (Table 5). However, final body weight differed significantly among dietary treatments (Figure 1). Shrimp fed RZ80 (8.00 ± 0.59 g) and PA20 (8.25 ± 0.86 g) achieved the highest final body weights. RZ40 (7.67 ± 0.46 g) and RZ60 (7.62 ± 0.66 g) produced intermediate responses, whereas CTRL (7.48 ± 0.71 g), RZ20 (7.51 ± 0.90 g), and PA10 (7.39 ± 1.18 g) resulted in the lowest final body weights and did not differ significantly from one another (*p* > 0.05). These findings indicate that, under intensive clear‐water conditions, diets supplemented with 800 mg/kg Ronozyme ProAct (RZ80) and 200 mg/kg ProAct 360 (PA20) resulted in significantly higher final body weights compared with the other treatments.

**Figure 1:**
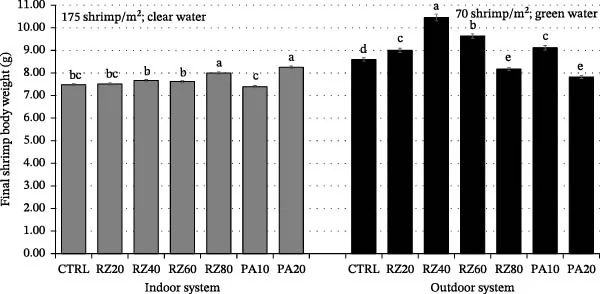
Final body weight (mean ± standard error) of juvenile *Penaeus vannamei* fed diets supplemented with graded levels of Ronozyme ProAct and ProAct 360. Different letters indicate significant differences among dietary treatments within each culture system according to Tukey’s HSD test (*p* < 0.05). Initial stocking densities were 175 and 70 shrimp/m^2^ in indoor and outdoor tanks, respectively. CTRL, control diet; RZ20, RZ40, RZ60, and RZ80: diets supplemented with 200, 400, 600, and 800 mg/kg Ronozyme ProAct, respectively; and PA10 and PA20: diets supplemented with 100 and 200 mg/kg ProAct 360, respectively.

**Table 5:** Growth performance, survival, feed utilization, and yield of juvenile *P. vannamei* fed graded levels of Ronozyme ProAct and ProAct 360 in indoor and outdoor culture systems.

Diets	Initial body weight (g)	Final survival (%)	Gained yield (g/m^2^)	Growth (g/week)	AFI (g/shrimp)	FCR
Performance—Indoor tanks—Clear water—High density						
CTRL	1.03 ± 0.09	92.6 ± 7.2	1034 ± 39	0.54 ± 0.06	10.9 ± 0.6	1.88 ± 0.24
RZ20	1.04 ± 0.09	95.3 ± 3.0	1073 ± 39	0.55 ± 0.07	10.8 ± 0.6	1.80 ± 0.31
RZ40	1.05 ± 0.07	92.6 ± 6.1	1061 ± 33	0.56 ± 0.04	10.7 ± 0.4	1.79 ± 0.25
RZ60	1.04 ± 0.07	92.1 ± 4.1	1049 ± 34	0.55 ± 0.06	10.8 ± 0.4	1.83 ± 0.25
RZ80	1.03 ± 0.09	91.6 ± 5.5	1104 ± 38	0.53 ± 0.21	10.4 ± 2.2	1.68 ± 0.40
PA10	1.04 ± 0.08	94.0 ± 3.2	1036 ± 53	0.53 ± 0.10	10.6 ± 0.7	1.87 ± 0.50
PA20	1.02 ± 0.09	91.6 ± 4.9	1114 ± 36	0.61 ± 0.07	10.9 ± 0.6	1.69 ± 0.19
Mean ± sd	1.04 ± 0.08	92.9 ± 5.0	1068 ± 143	0.55 ± 0.10	10.7 ± 0.9	1.80 ± 0.32
ANOVA *p*	0.921	0.476	0.528	0.560	0.773	0.618
Performance—Outdoor tanks—Green water—Regular density						
CTRL	0.50 ± 0.03	98.0 ± 2.0	558 ± 89^a^	0.81 ± 0.13^a^	13.7 ± 1.8	1.51 ± 0.09^ab^
RZ20	0.52 ± 0.03	98.4 ± 1.5	599 ± 100^a^	0.86 ± 0.15^a^	14.5 ± 1.8	1.50 ± 0.12^ab^
RZ40	0.51 ± 0.03	98.0 ± 1.6	767 ± 124^b^	1.11 ± 0.17^b^	15.7 ± 2.8	1.49 ± 0.04^ab^
RZ60	0.51 ± 0.03	98.1 ± 2.1	666 ± 70^ab^	0.96 ± 0.11^ab^	15.9 ± 1.1	1.48 ± 0.12^ab^
RZ80	0.51 ± 0.03	97.6 ± 1.9	576 ± 145^a^	0.84 ± 0.20^a^	14.7 ± 2.4	1.61 ± 0.13^ab^
PA10	0.51 ± 0.03	98.1 ± 1.6	608 ± 81^ab^	0.88 ± 0.12^ab^	14.5 ± 1.4	1.47 ± 0.08^a^
PA20	0.51 ± 0.03	97.6 ± 2.4	560 ± 109^a^	0.81 ± 0.15^a^	14.6 ± 2.0	1.65 ± 0.15^b^
Mean ± sd	0.51 ± 0.03	97.9 ± 1.9	—	—	14.7 ± 1.9	—
ANOVA *p*	0.605	0.979	0.009	0.009	0.335	0.005

*Note:* Values presented as means (±sd). Different letters in columns indicate statistically significant differences between diets within each culture system according to Tukey’s HSD test (*p* < 0.05). Initial stocking density: 175 and 70 shrimp/m^2^ in indoor and outdoor tanks, respectively. CTRL, control; RZ20, RZ40, RZ60, and RZ80: 200, 400, 600, and 800 mg/kg Ronozyme ProAct; and PA10 and PA20: 100 and 200 mg/kg ProAct 360, respectively.

In the outdoor green‐water culture system (70 shrimp/m^2^), temperature, salinity, and pH reached 29.1 ± 0.5°C, 16.0 ± 0.9 ppt, and 8.4 ± 0.2, respectively. Dietary protease supplementation significantly improved gained yield, growth rate, and FCR relative to CTRL (*p* < 0.01). The RZ40 diet increased yield and growth rate by 37.5% and 37.0%, respectively, compared with CTRL while improving FCR by 9.1%. Diets containing RZ20 (200 mg/kg) and PA10 (100 mg/kg) produced smaller but positive improvements in performance, whereas higher inclusion levels (RZ80 at 800 mg/kg and PA20 at 200 mg/kg) did not confer additional benefits. Survival and AFI were not affected by dietary treatment. Final body weight differed among treatments (*p* < 0.05). Shrimp fed RZ40 achieved a 21.6% higher final weight than CTRL, while RZ60 (600 mg/kg) and RZ20 (200 mg/kg) increased the final weight by 11.9% and 4.7%, respectively. In contrast, shrimp fed RZ80 and PA20 exhibited final weights of 5.0% and 9.1% lower than CTRL.

### 3.3. ADCs

In the digestibility system, mean water temperature, salinity, and pH reached 27.5 ± 0.7 and 28.2 ± 0.4°C, 13.6 ± 0.9 and 16.2 ± 0.9 ppt, and 7.7 ± 0.2 and 8.0 ± 0.2, for the standard and high SBM digestibility trials, respectively. Shrimp fed the standard SBM diets (33.66% SBM) exhibited similar growth and survival regardless of protease supplementation (Table 6). The initial body weight averaged 7.79 ± 0.23 g, increasing to a final body weight of 16.80 ± 1.14 g after the trial. Growth rate averaged 0.82 ± 0.10 g/week, survival 97.9 ± 4.0%, and gained yield 519 ± 70 g/m^2^, with no significant differences among dietary treatments (*p* > 0.05). However, shrimp fed the RZ40 diet consumed significantly more feed (25.1 ± 0.9 g/shrimp) than those fed the CTRL (22.9 ± 1.1 g/shrimp) or PA10 (23.0 ± 0.5 g/shrimp) diets (*p* < 0.0001), while FCR remained high but statistically unchanged (2.78 ± 0.35).

**Table 6:** Growth performance and feed utilization of juvenile *P. vannamei* fed protease‐supplemented diets during the digestibility trials.

Performance	Standard SBM diets			ANOVA *p*	High SBM diets			ANOVA *p*
	CTRL	RZ40	PA10		CTRL	RZ40	PA10	
Initial body weight (g)	7.86 ± 0.12	7.69 ± 0.29	7.83 ± 0.26	0.116	6.99 ± 0.56	7.04 ± 0.56	6.94 ± 0.54	0.273
Final body weight (g)	16.51 ± 1.37	17.12 ± 0.97	16.76 ± 1.09	0.359	27.76 ± 1.36	27.57 ± 0.80	27.85 ± 1.00	0.754
Survival (%)	98.2 ± 3.5	98.3 ± 3.4	97.2 ± 5.1	0.725	96.4 ± 5.4	95.6 ± 6.9	96.7 ± 4.2	0.852
Growth (g/week)	0.79 ± 0.13	0.86 ± 0.07	0.81 ± 0.10	0.171	2.05 ± 0.13	2.02 ± 0.07	2.06 ± 0.10	0.611
Gained yield (g/m^2^)	501 ± 78	550 ± 61	507 ± 71	0.134	1187 ± 123	1158 ± 120	1198 ± 78	0.589
AFI (g/shrimp)	22.9 ± 1.1a	25.1 ± 0.9^b^	23.0 ± 0.5^a^	<0.0001	29.9 ± 0.4^a^	30.5 ± 0.4^b^	30.1 ± 0.4^a^	<0.0001
FCR	2.80 ± 0.40	2.76 ± 0.25	2.77 ± 0.39	0.949	1.53 ± 0.18	1.60 ± 0.18	1.51 ± 0.10	0.290

*Note:* Means within a row sharing a common lowercase letter are not significantly different according to Tukey’s HSD test (*p* > 0.05). CTRL, control diet without protease supplementation; RZ40, diet with 400 mg/kg Ronozyme ProAct; and PA10, diet with 100 mg/kg ProAct 360. Standard and high SBM diets contained 33.66% and 51.82% (% of the diet, as‐is) SBM.

Abbreviations: AFI, apparent feed intake; FCR, feed conversion ratio.

For the 51.82% SBM diets (high SBM digestibility diets), performance responses were also consistent among dietary treatments (Table 6). The average initial and final body weights were 6.99 ± 0.55 g and 27.73 ± 1.05 g, respectively, with growth rates averaging 2.04 ± 0.10 g/week, survival 96.2 ± 5.5%, and gained yield 1181 ± 107 g/m^2^ (*p* > 0.05 for all parameters). As observed for the standard SBM diets, shrimp fed RZ40 consumed significantly more feed (30.5 ± 0.4 g/shrimp) compared to CTRL (29.9 ± 0.4 g/shrimp) and PA10 (30.1 ± 0.4 g/shrimp; *p* < 0.0001), but FCR values were statistically similar across treatments, averaging 1.55 ± 0.15.

In both standard and high SBM diets, protease supplementation markedly improved the ADCs of most nutrients and amino acids in juvenile *P. vannamei*. For the standard SBM diets (33.66% SBM digestibility diets), DM digestibility increased from 94.75 ± 1.05% in the CTRL to 95.99 ± 0.39% with RZ40 (*p* = 0.016). CP digestibility rose significantly from 67.80 ± 6.96% in CTRL to 78.16 ± 2.31% in RZ40 and 75.27 ± 8.38% in PA10 (*p* = 0.001). GE digestibility was also enhanced by protease addition, reaching 74.56 ± 9.21%, which was significantly higher than CTRL (58.42 ± 13.45%; *p* = 0.001). Among EAAs, the digestibility of lysine, methionine, and arginine notably increased with RZ40, averaging 88.75 ± 1.57, 90.06 ± 2.16, and 85.96 ± 1.18%, respectively, compared with 82.92 ± 3.31, 82.85 ± 4.27, and 79.31 ± 4.27% in the CTRL. PA10 yielded comparable improvements for these amino acids and did not differ statistically from RZ40 (Table 7). The overall digestibility of total EAA rose from 76.18 ± 5.19% in CTRL to 84.09 ± 1.37% in RZ40 (*p* = 0.001) and 82.47 ± 6.23% in PA10 (*p* = 0.001). A similar improvement was observed for NEAAs, increasing from 78.61 ± 4.85% in CTRL to 84.16 ± 1.14% in RZ40 and 84.61 ± 5.29% in PA10 (*p* = 0.002). The combined AA digestibility (EAA + NEAA) increased from 77.51 ± 4.99% in CTRL to 84.13 ± 1.24% in RZ40 and 83.64 ± 5.72% in PA10 (*p* = 0.001).

**Table 7:** Apparent digestibility coefficients (ADCs, %) of nutrients, energy, and amino acids in diets containing standard (33.66%) and high (51.82%) soybean meal levels supplemented with protease.

Nutrient	Standard SBM diet			ANOVA *p*	High SBM diets			ANOVA *p*
	CTRL	RZ40	PA10		CTRL	RZ40	PA10	
DM	94.75 ± 1.05^ab^	95.99 ± 0.39^b^	95.72 ± 1.42^ab^	0.016	94.76 ± 1.32^a^	96.38 ± 0.35^b^	95.92 ± 0.30^b^	<0.0001
CP	67.80 ± 6.96^a^	78.16 ± 2.31^b^	75.27 ± 8.38^b^	0.001	72.40 ± 6.56^a^	81.34 ± 1.54^b^	78.98 ± 2.04^b^	<0.0001
EE	85.97 ± 2.65	88.75 ± 1.60	85.63 ± 4.86	0.055	77.41 ± 5.73^a^	84.87 ± 0.84^b^	85.21 ± 0.99^b^	<0.0001
GE	58.42 ± 13.45^a^	61.72 ± 4.71^a^	74.56 ± 9.21^b^	0.001	61.14 ± 9.74^a^	75.34 ± 3.72^b^	72.36 ± 2.38^b^	<0.0001
Essential amino acids (EAAs)								
Arg	79.31 ± 4.27^a^	85.96 ± 1.18^b^	85.08 ± 5.23^b^	<0.0001	84.27 ± 3.90^a^	90.53 ± 0.81^c^	87.09 ± 1.23^b^	<0.0001
His	72.15 ± 5.53^a^	80.24 ± 1.20^b^	78.88 ± 7.42^b^	0.002	74.86 ± 6.80^a^	85.64 ± 0.98^c^	81.02 ± 1.61^b^	<0.0001
Ile	75.55 ± 5.73^a^	83.34 ± 1.15^b^	81.77 ± 6.65^b^	0.002	79.05 ± 5.02^a^	85.89 ± 0.91^b^	83.30 ± 1.42^b^	<0.0001
Leu	75.20 ± 6.16^a^	83.30 ± 1.46^b^	80.63 ± 7.10^ab^	0.003	72.64 ± 6.68^a^	81.38 ± 1.01^b^	77.42 ± 1.87^b^	<0.0001
Lys	82.92 ± 3.31^a^	88.75 ± 1.57^b^	87.39 ± 4.37^b^	<0.0001	86.05 ± 3.45^a^	90.74 ± 0.72^b^	89.24 ± 0.96^b^	<0.0001
Met	82.85 ± 4.27^a^	90.06 ± 2.16^b^	87.74 ± 4.87^b^	<0.0001	82.61 ± 4.25^a^	88.56 ± 0.27^b^	86.07 ± 1.39^b^	<0.0001
Met + Cys	82.80 ± 3.21^a^	88.05 ± 1.85^b^	86.08 ± 5.39^ab^	0.006	80.37 ± 4.71^a^	87.67 ± 0.88^c^	84.16 ± 1.49^b^	<0.0001
Phe	73.60 ± 6.26^a^	82.50 ± 1.11^b^	81.19 ± 6.41^b^	<0.0001	75.50 ± 5.93^a^	86.98 ± 0.97^c^	80.34 ± 1.69^b^	<0.0001
Thr	73.75 ± 5.84^a^	83.19 ± 2.01^b^	83.02 ± 5.99^b^	<0.0001	72.56 ± 6.91^a^	83.38 ± 0.92^c^	78.82 ± 1.62^b^	<0.0001
Val	67.76 ± 6.89^a^	77.74 ± 2.10^b^	74.46 ± 9.02^b^	0.003	73.44 ± 6.43^a^	82.72 ± 1.26^c^	78.68 ± 1.86^b^	<0.0001
Nonessential amino acids (NEAAs)								
Ala	67.84 ± 6.60^a^	76.76 ± 2.93^b^	74.91 ± 8.14^b^	0.004	70.26 ± 7.18^a^	80.52 ± 1.53^c^	75.49 ± 2.19^b^	<0.0001
Asp	86.93 ± 3.48^a^	88.11 ± 1.32^ab^	90.34 ± 3.59^b^	0.027	93.17 ± 1.67^a^	95.11 ± 0.53^b^	94.24 ± 0.57^b^	<0.0001
Cys	71.51 ± 9.77^a^	84.76 ± 1.90^b^	83.44 ± 6.21^b^	<0.0001	77.37 ± 5.34^a^	86.65 ± 1.71^c^	81.67 ± 1.97^b^	<0.0001
Gly	62.38 ± 10.90^a^	71.74 ± 2.53^b^	77.13 ± 7.58^b^	<0.0001	70.33 ± 7.64^a^	81.12 ± 1.77^c^	75.99 ± 2.27^b^	<0.0001
Glu	87.54 ± 2.89^a^	90.08 ± 0.67^b^	90.86 ± 3.28^b^	0.008	87.42 ± 3.21^a^	91.12 ± 0.67^b^	88.98 ± 1.07^a^	<0.0001
Hyd	—	—	—	—	—	—	—	—
Pro	77.89 ± 4.38^a^	85.46 ± 1.96^b^	83.04 ± 5.89^b^	0.001	74.58 ± 6.23^a^	89.66 ± 0.66^c^	78.54 ± 1.85^b^	<0.0001
Ser	72.00 ± 5.66^a^	79.73 ± 1.78^b^	78.80 ± 7.41^b^	0.003	72.59 ± 6.98^a^	82.55 ± 1.21^c^	77.40 ± 1.97^b^	<0.0001
Tau	32.12 ± 25.92^a^	57.75 ± 19.36^b^	24.59 ± 26.21^a^	0.005	90.82 ± 2.27^a^	95.33 ± 0.49^c^	56.99 ± 3.24^b^	<0.0001
Tyr	68.47 ± 8.43^a^	79.02 ± 1.86^b^	78.00 ± 7.38^b^	0.001	77.54 ± 5.82^a^	86.09 ± 1.71^b^	80.16 ± 2.05^a^	<0.0001
∑ EAA^1^	76.18 ± 5.19^a^	84.09 ± 1.37^b^	82.47 ± 6.23^b^	0.001	77.70 ± 5.48^a^	86.01 ± 0.90^c^	82.16 ± 1.52^b^	<0.0001
∑NEAA	78.61 ± 4.85^a^	84.16 ± 1.14^b^	84.61 ± 5.29^b^	0.002	79.83 ± 5.04^a^	87.36 ± 1.06^c^	83.05 ± 1.57^b^	<0.0001
EAA + NEAA	77.51 ± 4.99^a^	84.13 ± 1.24^b^	83.64 ± 5.72^b^	0.001	78.87 ± 5.24^a^	86.76 ± 0.99^c^	82.64 ± 1.54^b^	<0.0001

*Note:* Values represent means (±sd) of four measurements obtained from pooled samples collected from three to four replicate tanks, each stocked with 12 juvenile shrimp. Common lowercase letters within a line indicate no statistically significant differences in ADCs between diets, according to Tukey’s HSD test at the 0.05 significance level. CTRL, control diet without protease supplementation; RZ40, diet with 400 mg/kg Ronozyme ProAct; and PA10, diet with 100 mg/kg ProAct 360. Standard and high SBM diets contained 33.66% and 51.82% (% of the diet, as‐is) SBM.

Abbreviations: CP, crude protein; DM, dry matter; EE, ether extract; GE, gross energy.

^1^Tryptophan not included.

In the 51.82% SBM diets (high SBM digestibility diets), the effect of protease supplementation was even more pronounced (Table 7). DM digestibility increased from 94.76 ± 1.32% in CTRL to 96.38 ± 0.35% in RZ40 (*p* < 0.0001). CP digestibility rose from 72.40 ± 6.56 to 81.34 ± 1.54% with RZ40 (*p* < 0.0001), while EE digestibility improved from 77.41 ± 5.73 to 84.87 ± 0.84% (*p* < 0.0001). The ADC of GE increased by more than 20%, from 61.14 ± 9.74 to 75.34 ± 3.72% (*p* < 0.0001). For EAAs, RZ40 consistently produced the highest ADCs, particularly for lysine (90.74 ± 0.72%), methionine (88.56 ± 0.27%), and arginine (90.53 ± 0.81%), while the lowest values were recorded for the CTRL diet. The summed EAA digestibility improved from 77.70 ± 5.48% in CTRL to 86.01 ± 0.90% in RZ40 (*p* < 0.0001), with similar gains in NEAA digestibility (from 79.83 ± 5.04 to 87.36 ± 1.06%, *p* < 0.0001). The overall AA digestibility (EAA + NEAA) reached 86.76 ± 0.99% in RZ40, compared with 78.87 ± 5.24% in CTRL (*p* < 0.0001).

## 4. Discussion

### 4.1. Best‐Performing Doses of Protease in Indoor and Outdoor Systems

Our study demonstrated that dietary protease supplementation can enhance shrimp growth performance in both high‐density clear‐water and moderate‐density green‐water culture systems. Although the best‐performing supplementation levels differed between the two production systems, these responses should be interpreted cautiously because multiple environmental and management variables differed simultaneously between the indoor and outdoor trials, including water management strategy, natural food availability, stocking density, initial shrimp body weight, and culture duration. Consequently, the present study does not allow the independent effects of these variables to be separated but rather demonstrates that the best‐performing protease supplementation level may differ between contrasting culture conditions. In the indoor clear‐water system, where access to natural food sources was absent or minimal, shrimp required approximately twice the level of protease supplementation, that is, 800 mg/kg Ronozyme ProAct (47.5 ± 1.4 PROT/g) and 200 mg/kg ProAct 360 (76.7 ± 0.6 NFP/kg), to achieve maximum growth compared to the outdoor green‐water system, where lower levels (400 and 100 mg/kg, respectively) were sufficient.

A previous study has demonstrated that the whiteleg shrimp performs differently when raised under indoor and outdoor culture systems [35]. Under indoor clear‐water conditions (100 shrimp/m^2^), shrimp grew slower (1.00 ± 0.06 g/week) and showed lower survival (81.4 ± 7.3%) compared with those reared outdoors in green‐water tanks (60 shrimp/m^2^), which achieved 1.04 ± 0.09 g/week growth and 91.4 ± 5.4% survival. These differences were attributed to the limited natural food availability and higher stocking density in the indoor system, which imposed a more challenging environment despite greater control over water quality.

Rearing conditions, particularly stocking density, directly influence optimal nutrient levels in shrimp feeds. This relationship was clearly demonstrated by Façanha et al. [36], who showed that growth responses of juvenile *P. vannamei* to dietary methionine were density‐dependent. Shrimp reared at 50 animals/m^2^ achieved maximum growth (1.56 ± 0.04 g/week) with a dietary methionine level of 0.62% (DM basis), whereas higher stocking densities (75–100 animals/m^2^) required increased methionine concentrations (0.72%–0.81%) to sustain comparable growth performance. Methionine levels above these ranges did not result in further growth improvements, indicating that increased stocking density elevates amino acid demands, likely due to reduced natural food availability and greater metabolic competition.

In agreement with the study of Façanha et al. [36], our results also demonstrated that the need for protease supplementation is system‐dependent, with lower inclusion levels being sufficient under outdoor conditions. The superior performance observed at reduced enzyme inclusion levels in the outdoor system may be partially explained by the combined contribution of exogenous proteases supplied by the diet and those associated with nutrients derived from natural food sources. Moss et al. [37] found that the specific activities of serine protease, collagenase, amylase, cellulase, lipase, and acid phosphatase were significantly higher in shrimp reared in pond water than in those cultured in well water. The authors attributed the higher digestive enzyme activity in pond‐reared shrimp to the natural productivity of the pond environment, which serves as a source of organic substrates. This hypothesis is further supported by studies showing increased digestive enzyme activities in fish and shrimp raised in systems rich in natural food. For instance, Yu et al. [38] observed elevated digestive enzyme activities in *Carassius auratus* cultured in biofloc systems, while Xu et al. [39] reported higher specific activities of protease, amylase, cellulase, and lipase in shrimp exposed to bioflocs compared with those maintained in clear water, resulting in improved growth and weight gain. Therefore, in culture systems with natural food availability, satisfactory shrimp growth performance can be achieved through the combined effect of dietary exogenous proteases and those derived from natural food sources. This combination may contribute to an optimal presence of proteases in the shrimp digestive tract, complementing endogenous enzyme production and making higher enzyme inclusions unnecessary in outdoor systems.

It is also evident that higher protease inclusion levels may become counterproductive in green‐water conditions, as observed in the outdoor tanks where shrimp growth decreased at the highest enzyme supplementation levels. This phenomenon supports the idea that natural food‐derived enzymes complement moderate exogenous supplementation, whereas excessive dietary enzyme inclusion can lead to flat or even negative effects. Similar findings have been reported in broilers, where increasing protease doses resulted in significant reductions in growth performance and apparent CP digestibility in nutritionally adequate corn–soybean diets [40]. Excessive protease supplementation in poultry diets has been shown to interfere with gastric and intestinal enzyme activity, causing detrimental effects such as reduced growth performance, suppression of endogenous enzyme secretion, and lower digestible amino acid intake [40–42]. In fish, the effects of dietary protease are dose‐dependent, with low to moderate inclusion levels often improving nutrient utilization under limiting dietary conditions, whereas excessive inclusion levels can impair growth performance and intestinal health [43–45]. These observations are consistent with the present study, suggesting that in outdoor systems, inclusion levels of 400 mg/kg (19.8 ± 1.1 PROT/kg) of Ronozyme ProAct and 100 mg/kg (41.2 ± 0.1 NFP/kg) of ProAct 360 may yield the best results in terms of growth, yield, and FCR. Likewise, Liu et al. [44] demonstrated that a coated, heat‐stable neutral protease exhibited a clear dose‐response effect on growth performance in juvenile *Carassius auratus gibelio*, with regression analysis of weight gain rate identifying 400 mg/kg as the best‐performing dietary inclusion level, beyond which no further performance gains were achieved. Our observed best‐performing supplementation levels of Ronozyme ProAct, ranging from 400 to 800 mg/kg (outdoor and indoor, respectively), also align with the findings of Hasanthi et al. [22], who reported enhanced growth performance in *P. vannamei* fed both high‐fishmeal (20% FML, % of the diet, as‐is) and low‐FML (10%) diets. The authors reported that at 400 and 800 mg/kg protease supplementation, shrimp achieved higher final body weights and lower FCR comparable to those receiving high‐FML control diets. Santigosa et al. [21] reported that supplementation with ProAct 360 at 200 and 300 mg/kg to a diet containing 35% SBM and 2% fishmeal resulted in better feed intake and FCR in juvenile *P. vannamei* than supplementation at 100 mg/kg, although it did not improve final shrimp body weight.

Collectively, these results indicate that the production environment can either enhance or limit enzyme activity, and understanding these interactions is essential for defining protease inclusion levels that elicit favorable biochemical and physiological responses in *P. vannamei*.

### 4.2. Effect of Protease on Dietary Nutrient Digestibility

In the digestibility trials, apart from feed intake, no significant differences in shrimp performance were detected following dietary supplementation with 400 mg/kg of Ronozyme ProAct or 100 mg/kg of ProAct 360. In digestibility systems, shrimp are typically overfed to stimulate fecal production, a condition that can confound performance measurements. The observed increase in feed intake may reflect enhanced palatability or improved digestive kinetics resulting from the partial hydrolysis of dietary proteins, which elevates free amino acid concentrations in the feed matrix. Similar responses have been reported in *P. vannamei* where enzyme supplementation enhanced nutrient release and digestive efficiency without necessarily leading to measurable gains in growth performance [21, 22, 46].

Despite the absence of growth differences, protease supplementation significantly improved dietary nutrient digestibility regardless of the inclusion of SBM. ADCs for DM, CP, and energy were consistently higher in protease‐supplemented diets compared to the control in both standard and high SBM formulations. The dietary supplementation of protease promotes proteolytic and hydrolytic reactions that cleave complex protein molecules into smaller peptides and free amino acids [47], thereby increasing nutrient availability and improving metabolic utilization, as also evidenced in the dose‐response evaluation. Furthermore, proteases appear to play regulatory roles in enzymatic cascades by converting inactive zymogens into active enzymes [48]. In this work, protease supplementation also improved the digestibility of EE and GE, particularly in the high SBM diets, where lipid and energy ADCs increased by 8%–14% points relative to the control. This suggests that protease activity may have indirectly supported lipid digestion by stimulating other metabolic pathways, as proteases are known to activate zymogens involved in lipid and carbohydrate metabolism [48].

The digestibility of essential and NEAAs followed a consistent trend, with both protease‐supplemented diets showing significantly higher ADCs compared with the control. Improvements were especially evident for limiting amino acids such as methionine and lysine, whose ADCs increased from 82.9%–82.8% in CTRL to 88.8%–90.1% with RZ40 and 87.4%–87.7% with PA10 in standard SBM diets and from 86.1%–82.6% in CTRL to 90.7%–88.6% with RZ40 and 89.2%–86.1% with PA10 in high SBM diets. This is particularly relevant in high SBM diets, where SBM constituted 51.82% of the formulation and plant ingredients accounted for 81.03% of total composition. Protease supplementation thus compensated for the lower digestibility typically associated with plant proteins, which are deficient in sulfur amino acids and contain antinutritional compounds such as phytic acid, protease inhibitors, and β‐conglycinin [21, 49, 50]. Previous studies have shown that exogenous proteases can eliminate the adverse effects of antinutritional factors, improve the amino acid profile of the diet, and enhance energy utilization, leading to increased growth in rainbow trout (*Oncorhynchus mykiss*; [51]).

Cumulative digestibility of essential (ΣEAA) and nonessential (ΣNEAA) amino acids was also improved by both proteases. In high SBM diets, ΣEAA increased from 77.7% in the control to 86.0% with Ronozyme ProAct and 82.2% with ProAct 360, while ΣNEAA rose from 79.8% to 87.4% and 83.1%, respectively. These results confirm that exogenous proteases are effective in enhancing amino acid availability from both animal and plant sources, consistent with findings from rainbow trout [51] and other aquaculture species [43, 52].

Our results are consistent with Santigosa et al. [21]. The authors tested ProAct 360 at 100, 200, and 300 mg/kg (30,000–90,000 NFP/kg) and reported a clear improvement in digestibility at all inclusion levels. Protease supplementation significantly increased DM and CP ADCs, with no meaningful difference among the three doses, indicating that the digestive response plateaued at lower levels. Most amino acids also showed higher digestibility across all enzyme inclusions, except for isoleucine and leucine, which were unaffected. Improvements were consistent and dose‐independent, demonstrating that even the lowest inclusion was sufficient to enhance the amino acid release from the diet. Beyond growth and nutrient utilization, improved protein digestibility has important environmental implications for shrimp farming. Higher protein and amino acid digestibility reduces the fraction of dietary nitrogen excreted into the culture system as dissolved or particulate waste. A recent work by Santigosa et al. [21] demonstrated that enhanced protein digestibility through protease supplementation significantly reduced nitrogen excretion in shrimp, contributing to improved water quality and lower environmental loading. Therefore, the digestibility improvements observed in the present study may also translate into reduced nitrogen outputs, particularly in intensive systems where protein inputs are high.

Our results indicated that the response to exogenous protease supplementation depended on diet composition, particularly the relative contribution of plant‐ and marine‐derived protein sources in each formula. Plant‐based ingredients such as SBM and corn gluten meal (high SBM diets) contain protein fractions that are less accessible to endogenous shrimp proteases due to their structural complexity and association with antinutritional factors, whereas marine ingredients generally provide more digestible proteins and free amino acids that are readily hydrolyzed (moderate SBM diets). Consequently, diets with higher inclusion of SBM and other plant proteins tended to show a greater response to protease supplementation than diets with higher levels of fishmeal.

Overall, the data demonstrate that protease supplementation improved nutrient digestibility across diets, particularly in high–plant protein formulations, without adversely affecting growth performance. The findings of this study are consistent with the literature, highlighting the advantages of using exogenous proteases and demonstrating their ability to provide benefits such as improved FCR, higher weekly weight gain, and greater apparent nutrient digestibility [21–23, 46, 52, 53]. The observed benefits reinforce the role of exogenous proteases as functional additives in shrimp feeds, particularly for formulations rich in plant ingredients.

## 5. Conclusion

This study demonstrates that dietary protease supplementation enhances nutrient digestibility and may support growth performance in *P. vannamei*, with the best‐performing inclusion levels depending on the production system. In the indoor clear‐water culture system evaluated in this study, characterized by high shrimp stocking density and limited natural productivity, higher inclusion levels of 800 mg/kg Ronozyme ProAct or 200 mg/kg ProAct 360 were associated with maximum shrimp growth. In contrast, in the outdoor green‐water culture system, which differed simultaneously in several environmental and management aspects, including moderate stocking density and the presence of natural food, lower inclusion levels of 400 mg/kg and 100 mg/kg, respectively, achieved comparable results. The consistent improvement in ADCs, particularly for protein and EAAs, reinforces the value of proteases in practical shrimp feed formulations containing low fishmeal levels and moderate to high SBM inclusion. Improved nutrient digestibility has the potential to increase nutrient utilization efficiency and may contribute to lower nitrogen losses and improved environmental sustainability in shrimp farming, although these outcomes were not directly evaluated in the present study.

## Author Contributions

**Alberto J. P. Nunes**: conceptualization, methodology, investigation, formal analysis, data curation, writing – original draft, supervision. **Thiago Soligo**: conceptualization, funding acquisition, project administration, review and editing. **Ester Santigosa**: conceptualization, interpretation of results, writing, review and editing. **Caio G. D. Gomes**: investigation, data collection, laboratory analyses, writing, review and editing.

## Funding

This work was fully funded by dsm‐firmenich.

## Disclosure

All authors reviewed and approved the final manuscript. The sponsor provided financial support for the study but did not influence the experimental execution, data collection, statistical analyses, interpretation of the results, or the decision to publish. Thiago Soligo and Ester Santigosa, as coauthors and employees of dsm‐firmenich, contributed to the scientific design, interpretation of the results, critical revision of the manuscript, and approval of the final version in accordance with their authorship responsibilities.

## Ethics Statement

All experimental procedures involving shrimp were conducted in accordance with applicable national legislation and institutional guidelines for the use of animals in research. Shrimp are aquatic invertebrates and are not covered by Brazilian Federal regulations requiring ethical committee approval for animals of the phylum Chordata, subphylum Vertebrata; therefore, formal approval from an Institutional Animal Care and Use Committee was not required for this study. No anesthetic, analgesic, or surgical procedures were performed during the study. All reasonable measures were taken throughout the experimental period to minimize stress and avoid unnecessary suffering, including appropriate stocking densities, continuous aeration, maintenance of optimal water quality, and regular monitoring of animal health.

## Conflicts of Interest

Thiago Soligo and Ester Santigosa are employees of dsm‐firmenich, which uses the protease products Ronozyme ProAct and ProAct 360 evaluated in this study in its commercial feed solutions. The other authors declare no conflicts of interest.

## Data Availability

The datasets generated and analyzed during the current study are available from the corresponding author upon reasonable request.
